# Involvement of Membrane GRP78 in Trophoblastic Cell Fusion

**DOI:** 10.1371/journal.pone.0040596

**Published:** 2012-08-09

**Authors:** Sarah Fradet, Sandra Pierredon, Pascale Ribaux, Manuella Epiney, Kazuo Shin Ya, Olivier Irion, Marie Cohen

**Affiliations:** 1 Department of Gynecology Obstetrics, Faculty of Medicine, Geneva, Switzerland; 2 Biomedicinal Information Research Center, National Institute of Advanced Industrial Science and Technology, Tokyo, Japan; VU University Medical Center, The Netherlands

## Abstract

**Background:**

Glucose-regulated protein 78 (GRP78) is highly expressed in first trimester cytrophoblastic cells (CTBs), especially in syncytiotrophoblast (STB). However, the role of GRP78 in these cells has never been investigated.

**Methodology/Principal Findings:**

In this study, we have examined the role of GRP78 in trophoblast fusion using the Bewo choriocarcinoma cell line as a model of cytotrophoblast fusion. Down regulation of GRP78 by siRNA or chemical inhibitors and use of antibodies against GRP78 in culture medium significantly decreased forskolin-induced fusion capacity of Bewo cells suggesting the involvement of membrane GRP78 in trophoblast fusion. GRP78 expression was also studied in preeclamptic (PE) CTBs which are known to have lower fusion capacity compared to control CTBs. Interestingly, despite the increase of GRP78 mRNA in PE CTBs, membrane GRP78 is significantly decreased in PE CTBs compared to control CTBs, suggesting that relocation of GRP78 from the endoplasmic reticulum to cell surface is probably altered in PE CTBs.

**Conclusions:**

Our results imply that membrane GRP78 could play an important role in syncytialisation. They also suggest that deregulation of GRP78 expression or relocation at cell surface might be involved in pregnancy complication associated with defective syncytialisation, such as preeclampsia.

## Introduction

Placenta is a transient, autonomous and multifunctional organ whose main role is to permit feto-maternal exchanges of gas and nutrients [1]. At the feto-maternal interface, trophoblast cells differentiate according to the villous or the extravillous pathway [2]. In the extravillous pathway, extravillous cytotrophoblastic cells (evCTBs) proliferate and differentiate into an invasive phenotype [2]. These cells invade decidual stromal compartments as well as spiral arteries of the decidua and the proximal third of the myometrium [3]. In the villous pathway, villous cytotrophoblastic cells (CTBs) remain in the foetal compartment and fuse to form the syncytiotrophoblast (STB) [4]. STB is a multinuclear tissue forming the outer surface of the foetal part of the placenta and is crucial throughout pregnancy [5]. Indeed, this layer exerts unique specialized functions such as hormone secretion and generation of an immunological barrier [6]. The mechanism involved in vCTB differentiation and fusion into the STB is still unclear. Purified mononucleated vCTB aggregate and fuse *in vitro* to form multinucleated STB. This process is induced by treatment with cAMP, or with agents which increase intracellular cAMP levels [7]. The *in vitro* syncytialisation of human primary vCTB to a STB phenotype has been arbitrarily divided into two stages: the morphological and the biochemical differentiation [8]. The initial stage is referred as morphological differentiation and is accompanied to the aggregation and fusion of vCTB to form syncytium. The second stage is referred as biochemical differentiation and is characterized by expression of genes involved in substrate transport, hormone secretion and other functions of fully differentiated STB.

Glucose-regulated protein of 78 kDa (GRP78) is an endoplasmic reticulum (ER) molecular chaperone that belongs to the heat shock protein 70 (HSP70) family (for a review, see [9]). The primary functions of GRP78 are related to its capacity to bind hydrophobic regions on nascent polypeptides in the ER and to its pivotal role in the signalling cascade producing the unfolded protein response (UPR) [10]. GRP78 expression can be stimulated by a variety of environmental and physiological stress conditions such as glucose starvation or hypoxia [11], [12]. GRP78 is well-known to reside inside the ER lumen. However, numerous recent studies show that this chaperone is also located at the membrane of cancer cells and cells undergoing ER stress [13] [12]. The mechanisms responsible for the translocation of this protein from the ER to the plasma membrane remain poorly understood [14]. GRP78 on the outer plasma membrane functions as a receptor for a wide variety of ligands [10] and several small proteins can bind to surface GRP78 and modulate proliferation [13]. Recently, we have demonstrated that trophoblastic GRP78 was mainly found on the cell surface where it colocalized with p53 [15]. This distribution pattern of GRP78 and p53 is surprising but reveals another common trait between CTBs and cancer cells [15].

GRP78 protein and autoantibodies were also found in plasma of pregnant women. Interestingly, these autoantibodies were significantly lower in plasma of first trimester pregnant women who will subsequently develop preeclampsia (PE) [16]. Since hypoxia and glucose starvation occur in the first trimester PE placenta, it would be expected that GRP78 is overexpressed in these cells. PE is a two-stage disease characterised by abnormal placentation and vascular remodeling and the subsequent maternal syndrome marked by endothelial injury and activation. This pathology is associated with defects in the invasive pathway [6] and in the STB formation [17]. Immunohistochemistry of first trimester trophoblast also revealed that GRP78 was highly expressed in STB [15]. Since PE is associated with defects in STB formation [17], [18] and knowing that GRP78 is present at the membrane of CTBs and is strongly expressed in STB, we have decided to further investigate the possible role of GRP78 at the cell surface in the CTB-STB fusion process called syncytialisation. Because of the crucial role of STB throughout pregnancy, an excess or lack of STB formation could have dramatic consequences for both the mother and the foetus. Thus, it is important to understand the mechanisms leading to its formation. For this purpose, we investigated the role of GRP78 in the cell-fusion-inducible BeWo cell line.

## Results

### Expression of GRP78 is upregulated by forskolin treatment in BeWo cells and primary CTBs

**Figure 1:**
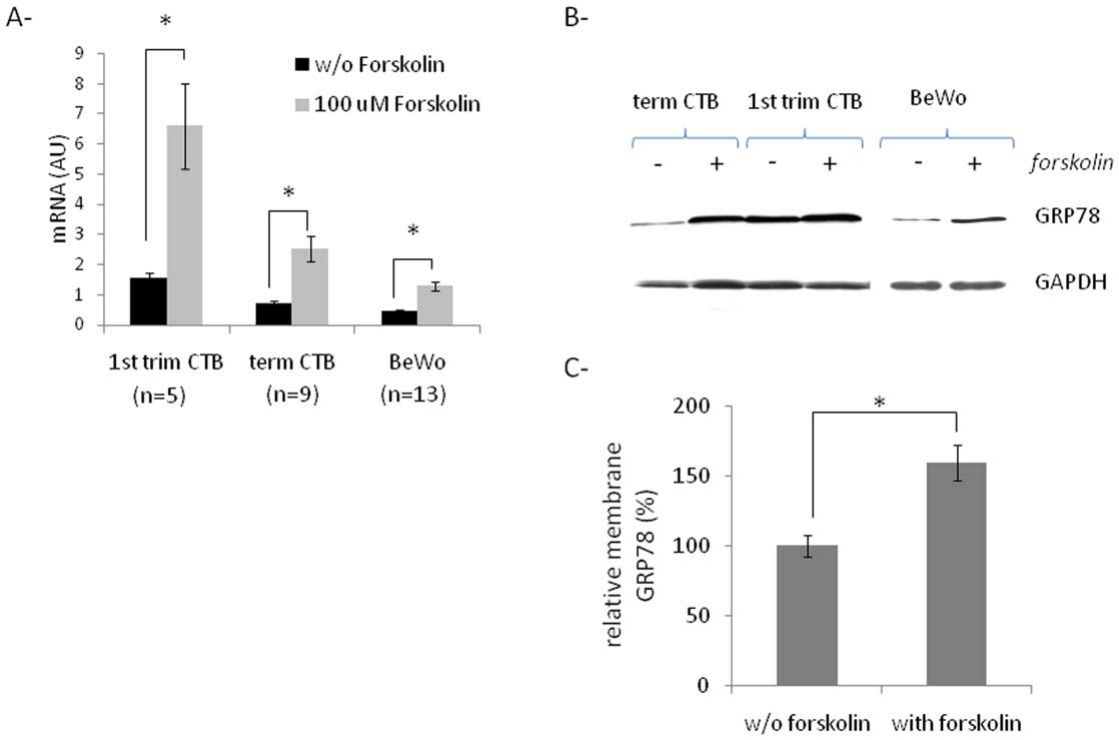
Effect of forskolin treatment on GRP78 expression in trophoblastic cells. Primary first trimester or term CTBs and BeWo cells were treated or not with 100 µM forskolin for 96 h or 48 h respectively. A- Total RNA was then extracted, reverse transcribe andGRP78 cDNA was quantified by qPCR and normalized to cyclophilin A. B- Proteins (40 ug) were analysed by western blot with anti-GRP78 and anti-GAPDH antibodies. C- Membrane GRP78 expression was quantified by cell-ELISA in BeWo cells. Results are presented as mean ± SEM. n = 3 * p<0.05.

Trophoblastic cells fuse in response to treatment with cAMP [7] [19]. Forskolin is often used to induce cAMP level and fusion of trophoblastic cells [19]. Therefore, we have first examined the effects of forskolin treatment on GRP78 expression in trophoblastic cells. As shown in figure 1, treatment of trophoblastic cells (primary first trimester and term CTBs as well as choriocarcinoma BeWo cell line) with forskolin led to a significant upregulation of GRP78 mRNA (A) and protein (B) expression. Moreover, forskolin-induced GRP78 expression led to increased membrane GRP78 expression in BeWo cells (figure 1C).

### Down-regulating GRP78 or blocking membrane GRP78 inhibits cell fusion

The choriocarcinoma BeWo cell line is the most extensively used cellular *in vitro* model to study villous trophoblast fusion [20]. This study took advantage of this cell line to investigate the role of membrane GRP78 in trophoblast cell fusion.

**Figure 2:**
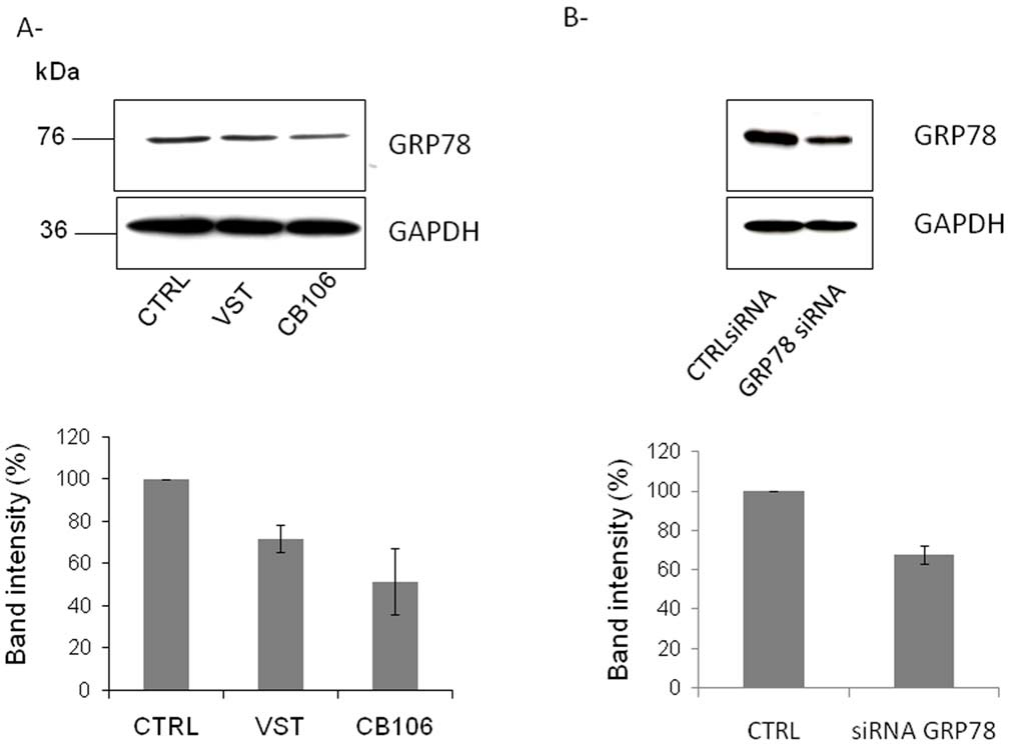
Downregulation of GRP78 in Bewo cells. A- Cells were untreated (CTRL) or treated with 20 µM VST or CB106 for 48 h. B- Cells were transfected with GRP78 or control siRNA for 48 h. A–B Upper panel: Immunoblots of GRP78 and GAPDH. Lower panel: The intensity of the GRP78 bands from three independent experiments was quantified and normalized to GAPDH.

**Figure 3:**
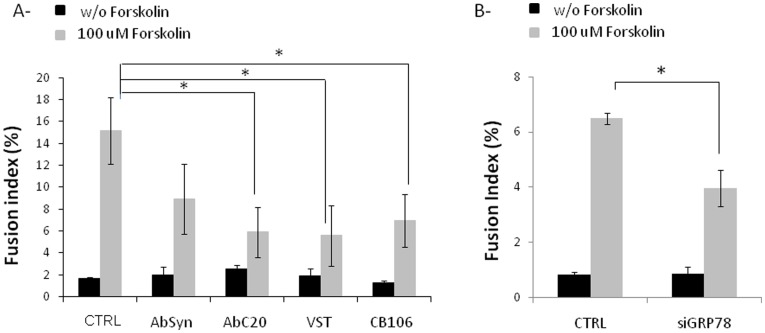
Role of GRP78 on fusion properties of BeWo cells. A- Cells were untreated (CTRL) or treated with anti-syncytin (AbSyn) or anti-GRP78 (AbC20) antibodies, 20 µM VST or CB106 for 48 h in the absence or presence of 100 µM forskolin. B- Cells were transfected with GRP78 or control siRNA for 48 h in presence or not of 100 µM forskolin. Fusion index was calculated for 3 independent experiments. * p<0.05.

We have first validated the efficiency of GRP78 down-regulation by a specific siRNA and two chemical inhibitors : versipelostatin (VST) [21] and CB106 (pimprinine). As shown in figure 2, the reduction of GRP78 protein expression was on average 30% with VST (figure 2A), 50% with CB106 (figure 2A) and 35% with GRP78 siRNA (figure 2B). The cell viability was only slightly decreased by the two down-regulators VST and CB106 (not shown). Fusion of cells was then measured in presence or not of down regulators of GRP78 or antibodies against GRP78 (AbC20).Treatment with antibodies against syncytin (AbSyn) known to inhibit fusion between trophoblast-derived cells [22] was used as positive control of decreased fusion capacity of BeWo cells. As shown in figure 3, down regulation of GRP78 by VST (figure 3A), CB106 (figure 3A) or GRP78 siRNA (figure 3B) significantly decreases forskolin-induced fusion of BeWo cells compared to control cells. Antibodies against syncytin and GRP78 (figure 3A) blocking syncytin and GRP78 membrane proteins respectively also decrease the fusion index of forskolin-treated BeWo cells. It would be noted that reverse transfection experiments has a negative impact on cell fusion which explains the difference of control values without forskolin between figure 3A and 3B.

**Figure 4:**
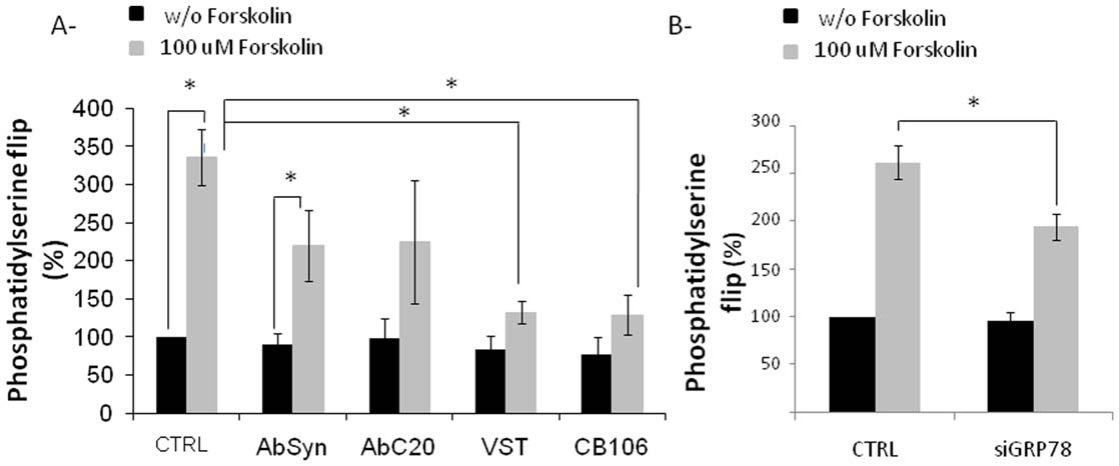
Role of GRP78 on phosphatidylserine flip of BeWo cells. A- BeWo cells were seeded on gelatin layer and treated or not with anti-syncytin (AbSyn) or anti-GRP78 (AbC20) antibodies, 20 µM VST or CB106 in the absence or presence of 100 µM forskolin for 48 h. B- BeWo cells were transfected with GRP78 or control siRNA and seeded on gelatin layer. The following day, cells were treated or not with forskolin for 48 h. Phosphatidylserine flip was evaluated by a colorimetric assay (APOPercentage). n = 3, * p<0.05.

Since exofacial phosphatidylserine was described as a necessary component for cell fusion [23] [24], we have also evaluated phosphatidylserine efflux in BeWo cells. Similar to the effect on the fusion index, chemical down-regulators of GRP78 (figure 4A) or GRP78 siRNA (figure 4B) significantly decrease the efflux of phosphatidylserine induced by forskolin compared to control cells. Antibodies against syncytin and GRP78 seem to slightly decrease forskolin-induced efflux of phosphatidylserine compared to control cells (figure 4A). However, these results are not significant due to inter-experiments variability and proliferative effects of these antibodies.

### GRP78 down-regulation decreases forskolin-induced secretion of human chorionic gonadotrophin (hCG)

**Figure 5:**
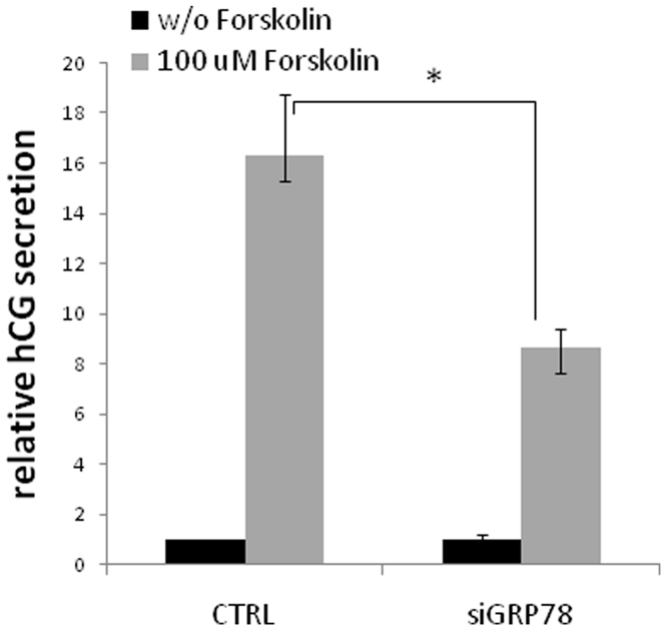
Effect of GRP78 down regulation on hCG secretion. BeWo cells were transfected with GRP78 or control siRNA and seeded on gelatin layer and then treated or not with 100 µM forskolin for 48 h. hCG level in culture supernatant was determined by ELISA and normalized to protein extract concentration. n = 3, * p<0.05.

Syncytialisation of cells is generally assessed by both cell fusion into syncytial unit and by induced secretion of hCG [5]. Indeed, differentiation of trophoblasts into syncytia is associated with increased production of hCG which can act in an autocrine manner to increase syncytium formation [25] [19]. We have thus measured hCG secretion in forskolin-induced BeWo cells transfected with GRP78 or control siRNA. As shown in figure 5, down-regulation of GRP78 expression by siRNA in BeWo cells significantly decreases the forskolin-induced secretion.

### Expression of membrane GRP78 in preeclamptic CTBs compared to control CTBs

PE has been already associated with defect of syncytialisation [26] [27]. We have thus evaluated expression and localization of GRP78 in control and PE CTBs. Table 1 reports the clinical information of women involved in this study. PE women had significantly elevated blood pressures and delivered earlier lower birthweight babies compared to normotensive patients (CTRL). Mean maternal age, parity and BMI were not significantly different between the 2 groups of patients.

**Table 1:** Demographic and clinical characteristics of the study groups.

	CTRL (n = 9)	Severe PE (n = 6)	p value
Maternal age (year)	33.2+/−1.8	34.6+/−1.9	0.66
Gestational age at delivery (week)	37.2+/−0.9	29.4+/−1	0.0003
BMI	26.8+/−1.8	27.4+/−3.1	0.86
Max systolic blood	122.8+/−3.2	166.8+/−7.6	0.0003
Max diastolic blood	80.1+/−2.2	106+/−4.8	0.0006
Parity	0.9+/−0.3	0	0.08
Gravidity	2.5+/−0.5	1	0.04
Baby weight (gram)	3021+/−299	1168+/−227	0.002

**Figure 6:**
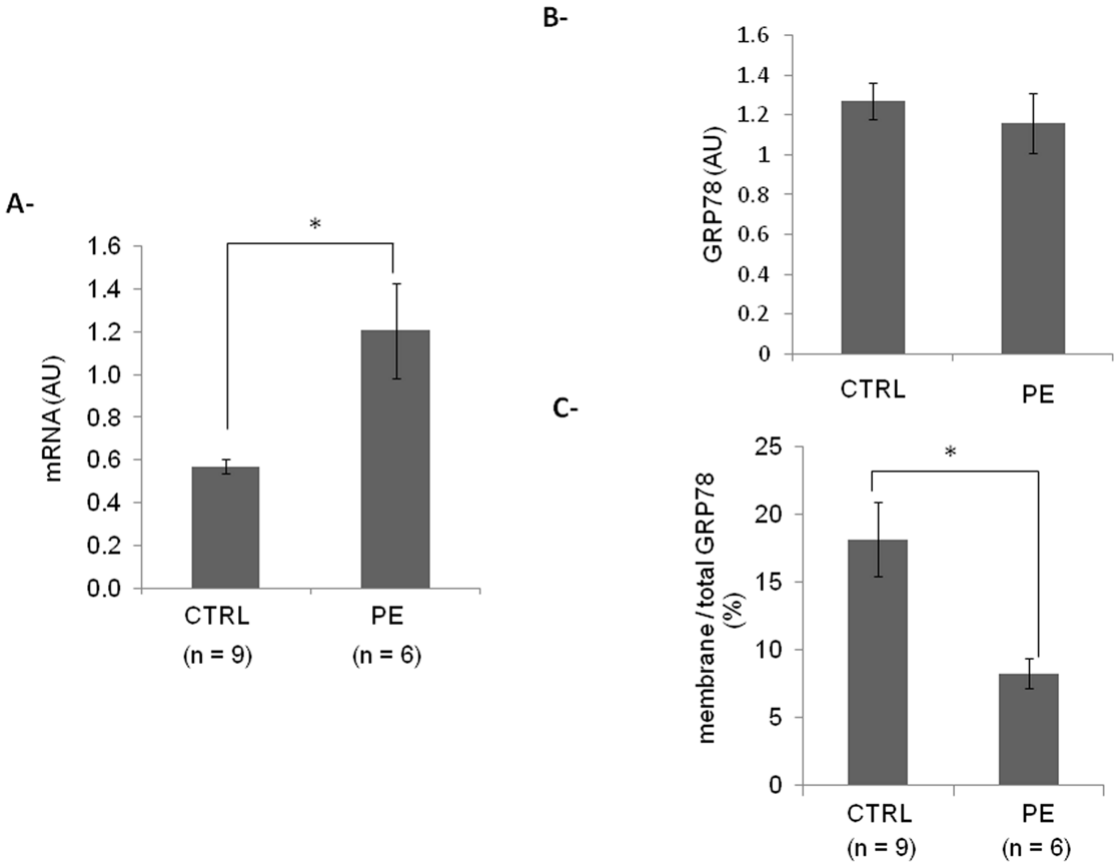
GRP78 expression in trophoblastic cells. **A-** GRP78 mRNA from control (CTRL) and preeclamptic (PE) trophoblastic cells was quantified by qPCR and normalized to cyclophlin A. **B-** GRP78 expression from control (CTRL) and preeclamptic (PE) trophoblastic cells was quantified by Cell-ELISA. **C-** Relative proportion of membrane GRP78 in trophoblastic cells purified from control (CTRL) and preeclamptic (PE) trophoblastic cells was evaluated by Cell-ELISA. Results are presented as mean ± SEM. n = 3 * p<0.05; AU: arbitrary unit.

Although GRP78 mRNA is significantly increased in PE CTBs compared to control cells (figure 6A), protein level of GRP78 measured by Cell-ELISA and confirmed by western blot (not shown) is not significantly different between control and PE cells (figure 6B). Interestingly, the proportion of GRP78 present at cell surface is significantly reduced in PE CTBs compared to control cells (figure 6C).

## Discussion

Our previous investigation showed high GRP78 expression in villous CTBs, and particularly in STB [15] [16]. Here, we have found that GRP78 mRNA and protein expression are increased in first trimester CTB compared to term CTB suggesting the importance of GRP78 during embryo implantation. We had previously suggested its involvement in trophoblastic invasion [15], but its role in STB was never investigated. GRP78 expression is regulated by various stimuli such as hypoxia or glucose starvation [12] and can also be stimulated by cAMP [28]. cAMP has long been known to be a promoter of trophoblastic cell fusion by regulating several proteins such as syncytin 1 and 2. In the present study, treatment of BeWo cells with forskolin, used as inducer of cAMP, led to upregulation of GRP78 expression, concomitant with an increase in cell fusion. Moreover, down regulation of GRP78 led to a defective BeWo cell fusion and biochemical differentiation, providing evidence that this protein is involved in syncytialisation of BeWo cells. As far as we know, among the long list of functions that have been attributed to GRP78, cell fusion had never been mentioned. Furthermore, the decreased capacity of BeWo cell fusion in the presence of anti-GRP78 antibodies strongly suggests that cell-surface GRP78 is a necessary component of trophoblastic cell differentiation. However, we have not yet identified a partner of GRP78 involved in this process and the mechanism of cell fusion linked to GRP78 remains to be investigated. Nevertheless, alpha 2 macroglobulin seems to be an interesting candidate if we consider signaling pathways involved in trophoblastic fusion [19], [29], [30] and signaling pathways activated by alpha 2 macroglobulin binding to membrane GRP78 described in cancer cells [14].

Several studies have highlighted abnormal trophoblastic differentiation in choriocarcinoma, PE, and a few other pathological conditions of pregnancy (for review, [8]). In PE, incomplete differentiation of CTBs to both invasive and villous CTBs was observed. As a consequence, there is shallow invasion of the pregnant uterus and defective syncytin expression in STB responsible for the characterized disturbances in STB in PE such as increased numbers of syncytial knots [31] [26].

In the present study, we have observed an apparent overexpression of GRP78 mRNA in PE CTBs which is consistent with the fact that GRP78 expression is induced by glucose deprivation and hypoxia, two characteristic features of PE. However GRP78 protein level is not modified in PE CTBs compared to control CTBs. This could be due to an increased turnover of GRP78 protein in PE CTBs compared to control CTBs. In contrast, membrane GRP78 level is decreased in PE CTBs compared to control ones. This last observation could explain the reduced level of circulating GRP78 autoantibodies found in pregnant women who subsequently developed PE [16]. Moreover, since GRP78 protein level is not different between PE CTBs and control CTBs, the decreased membrane GRP78 expression observed in PE CTBs is probably due to an altered GRP78 translocation from the ER to the plasma membrane. At present, the mechanism allowing GRP78 to translocate to the different cellular compartments are not well understood. Indeed, overexpression of GRP78 can lead to cell surface localisation by saturation of KDEL retention mechanism, but ER stress can also actively promote GRP78 surface expression [32]. Moreover, relocalization of GRP78 to cell surface involves cell specific transporting proteins such as MTJ-I in macrophages [33] or Par-4 in prostate cancer PC-3 cells [34], [35]. Therefore, the decreased expression of cell surface GRP78 in PE CTBs could be due to an altered expression of GRP78 trophoblastic transporting proteins. Mechanisms leading to impaired relocalisation of GRP78 at cell surface in PE cells have to be investigated to better understand the dysfunctions responsible for PE.

In conclusion, membrane GRP78 seems to be involved in trophoblastic cell fusion and biochemical differentiation *in vitro*. Moreover, we have highlighted a possible impaired mechanism of GRP78 relocation in PE CTBs which could be associated to defective syncytialisation *in vivo*. Together, these results strongly suggest the importance of membrane GRP78 in syncytialisation process.

## Materials and Methods

### Ethics statement

This research has been approved by the departmental ethics committee of maternity and pediatrics, University Hospital of Geneva (10-001 and 02-088). Informed written consent was obtained from all patients before their inclusion in the study.

### BeWo Cell culture

BeWo cells (ATCC, CCL-98) were kindly provided by Dr Thierry Fournier (Inserm U767, Paris, France) and cultured at 37 °C and 5% CO_2_ in Ham's F12K medium (Gibco, Invitrogen, Basel, Switzerland) supplemented with 10% FBS (Biochrom AG, Oxoid AG, Basel, Switzerland) and 0.05 mg/ml gentamycin (Invitrogen, Basel, Switzerland). Syncytialisation was induced 24 h after cells seeding of with 100 µM forskolin (Sigma-, St Louis, MO, USA) for 48 h. Control cells were treated with 1% ethanol (the vehicle for forskolin).

### Study groups

Severe PE was diagnosed using standard definitions from hypertension defined as a systolic blood pressure level ≥160 mmHg or a diastolic blood pressure level ≥110 mmHg on two occasions and proteinuria ≥3+ on a urine stick or ≥5 g in a 24-hour urine specimen (ACOG practice, 2002).

Nine control and 6 PE patients were recruited for this study. Their characteristics are given in Table 1.

### Purification of CTBs

CTBs were isolated from first trimester trophoblast obtained after legal abortion and placentas obtained after delivery as described previously [36]. In brief, fresh tissue specimen were isolated and washed several times in sterile HBSS. Tissue was then enzymatically digested 5 times for 20 min at 37°C (0.25% trypsin, 0.25 mg/ml Dnase I). After incubation, the trypsin cocktail was neutralized with FBS, and the cells resuspended in DMEM (Gibco, Invitrogen, Basel; Switzerland). This cell suspension was filtered (100 µm mesh), laid onto a Percoll gradient (70% to 5% Percoll diluted with HBSS) and centrifuged for 25 min at 1200 *g*. The 30–45% percoll layer containing CTBs was collected, the cells washed and resuspended in DMEM. Cells were then immunopurified with immobilized anti-CD45 antibodies

### Real-time quantitative PCR

RNA was extracted using RNeasy mini kit (QIAGEN, Basel, Switzerland). Reverse transcription was performed with 400 ng of total RNA using QuantiTect Reverse Transcription kit (QIAGEN). The quantitative detection of the PCR product was performed using the qPCR Mastermix Plus for SYBR Green I (Eurogentec, Seraing, Belgium), supplemented with fluorescein (Bio-Rad, Reinach, Switzerland), with the iCycler iQ System (Bio-Rad). The relative expression of GRP78 was normalized to the housekeeping gene cyclophilin A. Oligonucleotide primers for qPCR were as follows: human cyclophilin A forward 5′-TACGGGTCCTGGCATCTTGT-3′ and reverse 5′-CCATTTGTGTTGGGTCCAGC-3′, human GRP78 forward 5′-CGTGGAGATCATCGCCAAC-3′ and reverse 5′-ACATAGGACGGCGTGATGC-3′.

### siRNA transfection

Cells were plated at 3×10^5^ cells into 6-well plates or at 3×10^4^ cells into 96-well plates and immediately transfected with 40 nM of GRP78 siRNA (Santa Cruz Biotechnology, Labforce, Nunningen, Switzerland) or control siRNA (SiRNA-A, Santa Cruz Biotechnology). Transfection of siRNA was carried out using Lipofectamine 2000 (Invitrogen, Basel, Switzerland) following the reverse transfection protocol provided by the manufacturer.

### Western Blot

Whole BeWo cell extracts (40 µg of proteins) were fractionated by SDS-Page 10% and transferred to nitrocellulose membrane for immunoblot analysis using rabbit anti-GRP78 antibodies (GL-19, 1∶3000 dilution from Sigma) and mouse anti-GAPDH antibodies (1∶30 000 dilution from Millipore, Temecula, CA, USA).

### Fusion index

To visualize syncytia, BeWo cells were fixed in methanol at −20°C for 30 min and incubated in PBS containing 3% BSA for 30 min to eliminate non-specific binding. Cells were then incubated in the presence of goat anti-desmoplakin antibodies (1∶50 dilution, from Santa Cruz Biotechnology,) overnight at 4°C, washed with PBS and incubated with anti-goat IgG-HRP (1∶500 dilution, from Santa Cruz Biotechnology) for 1 h at room temperature. After washes in PBS, staining was revealed in diaminobenzidine (Dako).

The fusion index expressed in percent was calculated as follows: [(N-S)/T]×100, where N equals the number of nuclei in syncytia, S equals the number of syncytia and T equals the total number of nuclei counted. This index was calculated for 3 independent experiments, run in triplicate.

### Phosphatidylserine flip

Bewo cells were seeded on gelatine layer and treated with forskolin as mentioned before. Phosphatidylserine flip was evaluated in 3 independent experiments, run in triplicate by a colorimetric assay (APOPercentage from Biocolor Life Science, UK) following instructions of manufacturer.

### Cell-ELISA of membrane GRP78

Cell-ELISA was performed on CTBs as previously described [16]. Relative proportion of membrane GRP78 was calculated by dividing absorbance of membrane GRP78 (measured on non-permeabilized cells) over total GRP78 (measured on permeabilized cells) for 3 independent experiments run in triplicate.

### ELISA of hCG

BeWo cells were transfected with control or GRP78 siRNA and then cultured for 48 h in the presence or not of 100 µM of forskolin before culture medium was collected and centrifuged for 5 min at 14 000 g. Levels of hCG in supernatants were determined by enzyme immunoassay (ELISA; DRG International, Diagnostik Medizintechnik, Oberdof, Switzerland) following manufacturer's instructions and normalized by total cellular protein content of corresponding wells.

### Statistical analysis

Data were expressed as means ± SEM for n different samples. Statistical differences between samples were assessed by the Student's t test and the p value<0.05 was considered significant.
